# Computational Mapping Identifies Localized Mechanisms for Ablation of Atrial Fibrillation

**DOI:** 10.1371/journal.pone.0046034

**Published:** 2012-09-26

**Authors:** Sanjiv M. Narayan, David E. Krummen, Michael W. Enyeart, Wouter-Jan Rappel

**Affiliations:** 1 Department of Medicine and Veterans Affairs Medical Center, University of California San Diego, San Diego, California, United States of America; 2 Department of Physics and Center for Theoretical Biological Physics, University of California San Diego, San Diego, California, United States of America; Georgia State University, United States of America

## Abstract

Atrial fibrillation (AF) is the most common heart rhythm disorder in the Western world and a common cause of hospitalization and death. Pharmacologic and non-pharmacologic therapies have met with limited success, in part due to an incomplete understanding of the underlying mechanisms for AF. AF is traditionally characterized by spatiotemporally disorganized electrical activation and, although initiating triggers for AF are described, it is unclear whether AF is sustained by spatially meandering continuous excitation (re-entrant waves), localized electrical sources within the atria, or some other mechanism. This has limited therapeutic options for this condition. Here we show that human AF is predominantly caused by a small number (1.8±0.9) of localized re-entrant waves or repetitive focal beats, that remain stable with limited spatial migration over prolonged periods of time. Radiofrequency ablation that selectively targeted the sites of these sources was able to immediately terminate fibrillation and eliminate the arrhythmia with high success. Our results show that human AF, despite apparent spatiotemporal disorganization, is often perpetuated by a few spatially-constrained and temporally conserved sources whose targeted ablation can eliminate this complex rhythm disorder.

## Introduction

Heart rhythm disorders are defined by abnormal spatial or temporal patterns of electrical propagation. Fibrillation, the most prevalent human rhythm disorder, is characterized by extremely rapid, temporally irregular and spatially disorganized electrical activity with potentially disastrous consequences. In the ventricles, fibrillation causes an abrupt loss of cardiac output (sudden death), and is the leading cause of death world-wide [1]. Fibrillation of the atria affects over 10 million individuals in the U.S. and Europe [2], [3] in whom it contributes to heart failure, stroke and death. Despite numerous advances in therapy, AF is rarely cured [2]–[4] by defibrillating electrical shocks [2], [5] or anti-arrhythmic medications, both of which have modest long-term efficacy [2], [4]. Organized arrhythmias [6], [7], including the Wolff-Parkinson-White syndrome which contains an anatomically fixed reentry pathway [8], [9], exhibit rapid activity that can be destroyed by targeted ablation for a cure. However, this is not currently true for fibrillation.

Several mechanisms have been proposed for the maintenance and recurrence of AF, including electrical remodeling [10], [11], structural remodeling [12]–[14] and disturbed intracellular calcium homeostasis [15]. Seminal work by Haïssaguerre et al. has described initiating triggers for AF that can be eliminated by ablation [16] and genetic factors have been identified that contribute to the underlying propensity to AF [17], [18]. However, understanding the mechanisms that sustain AF, once triggered, is lacking [4], [19], such that current ablation procedures often fail to terminate AF and require electrical shock to restore sinus rhythm [4]. AF subsequently recurs in 30–50% of these patients within a 1-year period [4], [20] with little improvements in outcome in recent years [21].

In some animal models, reentrant spiral waves (rotors) may act as sources for fibrillation [22]–[25], and may be numerous [23], drift within the atria [26] or ventricles [23], or extinguish over time [23], [26], [27]. In other models, AF exhibits non-localized waves within the atrium [28]–[30]. Due to interspecies differences, including the rarity of spontaneous AF in animals and the fact that human AF is age-related and the product of co-existing diseases that are difficult to model, none of these mechanisms has yet been proven in human AF. Indeed, the existence of rotors is disputed in human AF [29], [31]. Similarly, focal beats, small regions of tissue from where activation emanates centrifugally and that may drive AF in animal models [31]–[33], are rarely identified *a priori* as sustaining mechanisms for human AF [29], [31].

**Figure 1:**
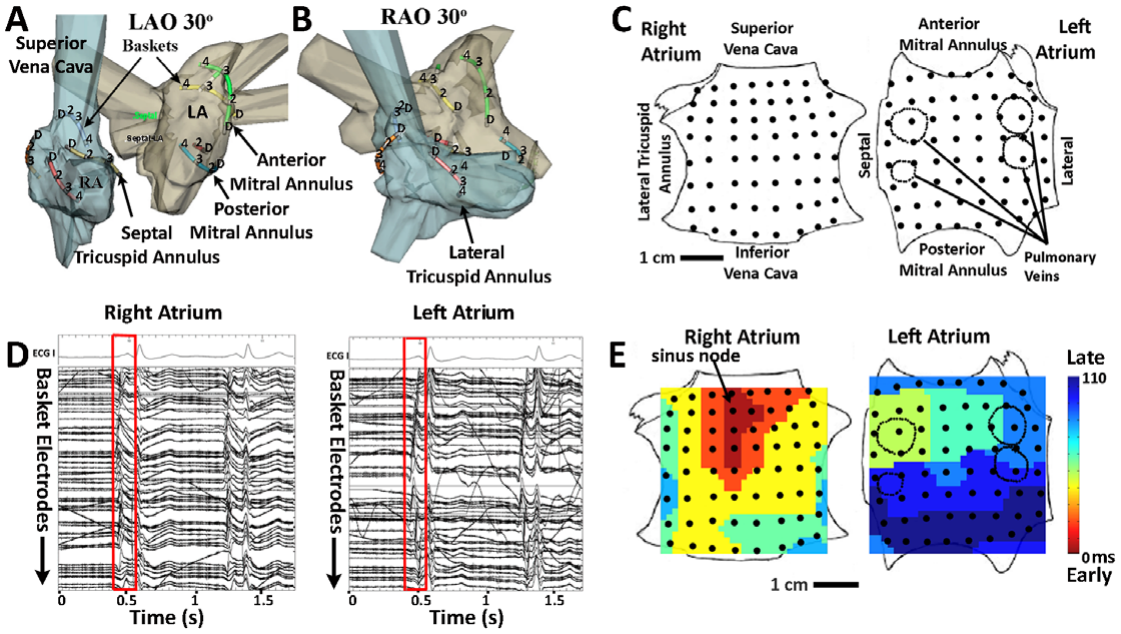
Electrical and Anatomic Mapping of Human Atria. **A**. Basket catheter splines are represented as colored lines registered within the right (blue) and left (grey) atria in left anterior oblique and **B**. right anterior oblique projections. The figure shows alternate splines with positions D (distal) to 4 representing electrodes 1 to 8. **C**. Bi-atrial schematic showing right atrium as if opened at its poles and left atrium as if opened at its equator, with electrode positions (black dots). **D**. ECG leads I, aVF and V1 and electrograms of one sinus rhythm beat (red box) captured by baskets in right and left atrium. **E**. Maps of sinus rhythm activation (isochrones) from high right atrium (sinus node) to low lateral left atrium created from bi-atrial basket recordings.

To define the predominant mechanisms underlying the perpetuation of AF in humans, we developed an approach to map human AF during minimally invasive procedures, combining high temporospatial resolution mapping of both atria (Fig. 1A–D) with patient-specific computational analyses of atrial activation and recovery.

We studied 80 subjects with a broad range of AF phenotypes including paroxysmal AF (defined as episodes that self-limit within 7 days), persistent AF (episodes that terminate only with cardioversion) or longstanding persistent AF (continuous AF for over 1 year) [4], and undergoing a first ablation as well as those who had failed conventional ablation (see Table S1 for patient characteristics). Details of our patient population and mapping technique are presented in the Methods and Supporting Information. Our mapping technique revealed localized sources (electrical rotors or focal beats) in almost all patients. Furthermore, we found that these sources were few in number and stable over prolonged periods of time that can be as long as months. These surprising findings enabled us to terminate AF with targeted ablation at the site of the localized sources. Finally, patients undergoing this targeted ablation experienced a superior rate of AF elimination in the long-term compared to patients undergoing traditional ablation procedures focusing on trigger mechanisms near the pulmonary veins.

## Results and Discussion

### Mapping of sinus rhythm

As a validation for our approach, we first computed activation patterns in patients during normal sinus rhythm using baskets in both the left and right atrium. This approach led to no adverse events in our experience. Fig. 1A–B depicts electrode locations registered within patient-specific atrial geometry, and shows that the baskets cover the vast majority of both atria. Fig. 1D shows the electrograms of one sinus rhythm beat in a patient, along with ECG leads. From this, a spatial map of normal sinus rhythm (isochrones) can be computed and shows activation from high right atrium (sinus node) to low lateral left atrium (Fig. 1E), consistent with known activation patterns.

### Rotors and focal sources are common during AF

**Figure 2:**
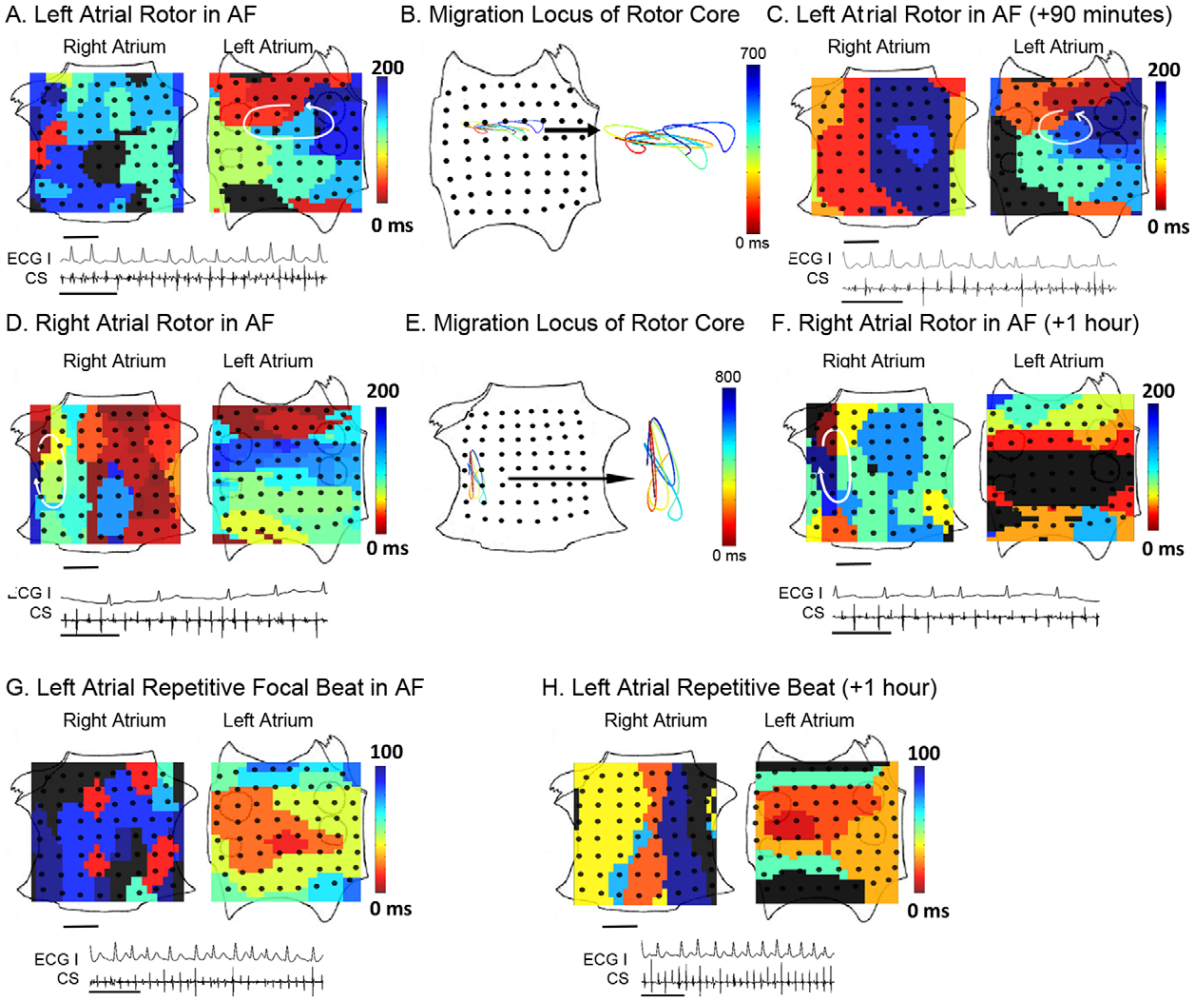
Stable Localized sources underlie human atrial fibrillation. **A**. Isochrones show an LA rotor in paroxysmal AF, with electrograms during AF (ECG lead I and CS electrodes; scale bar 1 second). Activation times are color-coded (black indicates non-activated, diastole). **B**. Spatially constrained migration locus of the rotational center, computed every 25–45 ms and joined using third-order Bézier curve fitting. **C**. Isochrones 90 minutes later, indicating temporal conservation of the rotor. **D**. Isochrones of a RA rotor in persistent AF. **E**. Migration locus. **F**. Isochrones 1 hour later. **G**. LA repetitive focal beat in paroxysmal AF. **H**. Conservation of focal beat 1 hour later. In each case, ablation only at the source locus terminated AF within <5 minutes. Scale bar 1 cm.

**Figure 3:**
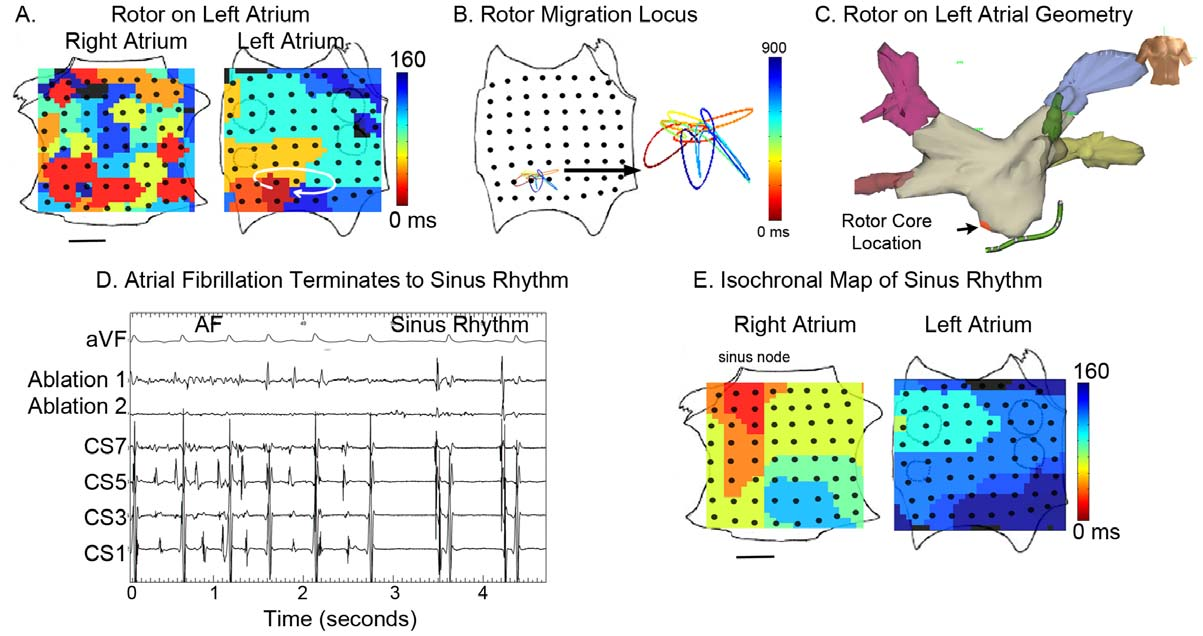
AF termination by ablation of Stable LA rotor. **A**. Left atrial rotor during paroxysmal AF visualized using isochrones. **B**. Migration locus of the rotational center, color-coded over time. **C**. Ablation lesions at rotor in low left atrium, applied 1 hour after initial recording of the rotor, shown on patient specific geometry. Red lesion is where AF terminated, and 3 other lesions (gray) were also applied. **D**. Electrode recordings during AF with termination to sinus rhythm by <1 minute ablation at the rotational center (ECG lead aVF, and electrodes at ablation catheter, coronary sinus). **E**. Isochronal map of sinus rhythm. The patient remains free of AF on implanted cardiac monitor at 9 months. Scale Bar 1 cm.

When applied to AF, our computational mapping technique revealed localized and stable sources in 96% of patients. This is in contrast to recent human studies in which localized sources for AF were rarely or never observed [29], [31]. Electrical rotors in AF, defined in this study as continuous sequential activation rotating around a central region, were revealed in 86% of patients (Fig. 2A–F and Table S2). Different computational approaches gave the same results on rotor location and characteristics (see Methods and compare fig. 3 with Figure S1).

Repetitive focal beats in AF, defined in this study as activation radiating from a source region, were revealed in 29% of patients (Fig. 2GH and Table S2). Although elegant studies suggest that focal beats in fibrillation may represent breakthrough from reentrant waves on the opposing cardiac surface [29], simultaneous epicardial mapping would be required to resolve differences across the cardiac wall. Analysis of the directionality of propagation across cycles confirmed that localized sources drive fibrillatory activity (Figure S2).

**Figure 4:**
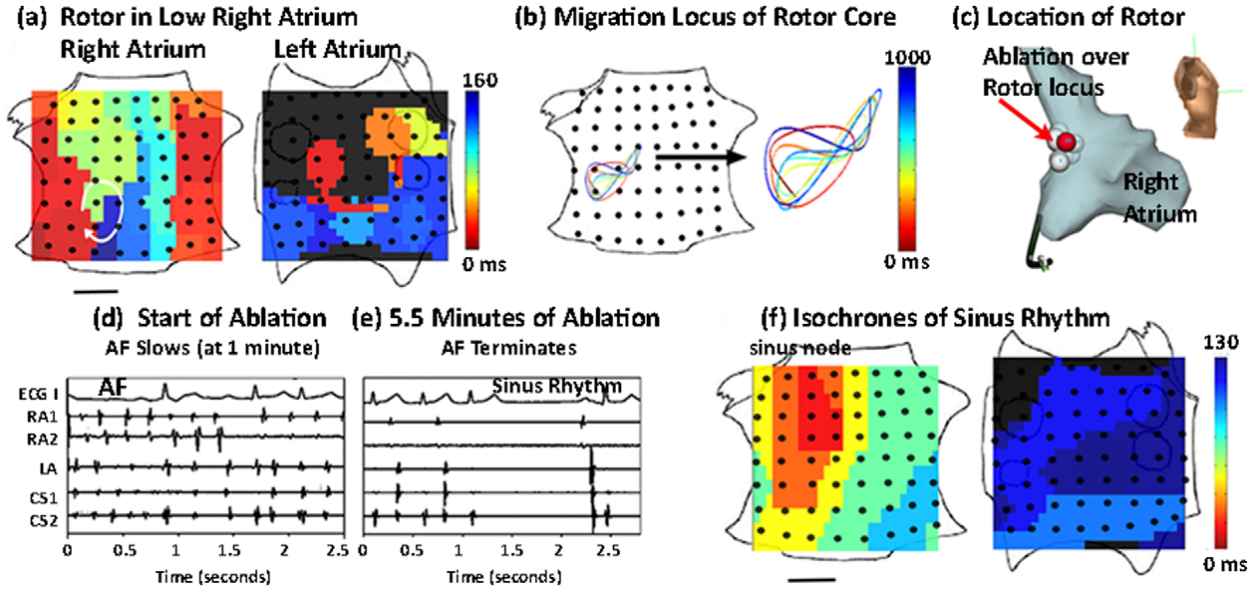
AF Termination by ablation of a Stable RA Rotor. **A**. Isochrones show a RA rotor and concurrent LA focal beat during persistent AF. **B**. Spatially constrained rotational center locus. **C**. Ablation lesions at lateral RA rotor on patient specific geometry (performed 2 hours after initial recording of rotor). A total of 11 lesions were applied (shown), with AF termination to sinus rhythm at 5.5 minutes. The red lesion indicates where ablation terminated AF. **D**. Electrograms AF terminating to sinus rhythm with localized ablation at rotor (total duration 5.5 minutes) (ECG lead I, intracardiac electrodes in RA, LA and CS). **E**. Isochrones of sinus rhythm. After ablation, the patient remains AF-free at 12 months on implanted cardiac monitor. Scale bar 1 cm.

Study patients often demonstrated more than one coexisting electrical rotor or repetitive focal beat (Fig. 4, Table S2), for an average of 1.8±0.9 for both atria. By comparison, single fibrillating sheep atria [26], that are substantially smaller than either human atrium, showed 1–3 sources. The number of coexisting sources was higher in patients presenting with advanced AF (i.e. persistent compared to paroxysmal AF, p<0.05), but was unrelated to whether patients were being studied for the first time or had previously failed conventional ablation (1.8±0.9 vs 1.8±0.6, respectively p = 0.84), patient age, left atrial diameter or duration of AF history.

### Localized sources are stable during AF

Computational mapping showed that rotational centers and focal beat origins in fibrillation were unexpectedly stable, migrating along circumscribed loci that partially overlapped between cycles (Fig. 2BE, 3B and 4B and Table S2) with areas 2.5±1.2 cm^2^ and 2.1±1.8 cm^2^, respectively. Notably, source locations were conserved between maps acquired 115±57 minutes apart throughout mapping (Fig. 2), showing that sources for human cardiac fibrillation are conserved for at least several hours. One patient presented the unusual opportunity to demonstrate conservation of a left atrial rotor during AF between procedures separated by 8 months (Figure S3). Conversely, rotors in animal models of AF may last for short periods before extinguishing [26]. Focal beats in human AF were also stable for hours (Fig. 4 and Table S2), while focal beats in animal models of AF were spatially variable and destabilized AF rotors [33]. This illustrates potential differences between AF in animals and the human condition.

### Targeted ablation of localized sources can eliminate AF

The spatially constrained and temporally conserved nature of a small number of rotors and focal beat sources in human AF presented an opportunity to establish their causal role via targeted elimination. We ablated the rotational center or origin of localized sources during ongoing AF by directly applying radiofrequency energy in 26 patients (of the population of n = 80), of whom 19 had persistent AF. Of 16 of 26 patients who exhibited 1 or 2 coexisting sources, ablation targeted to the limited migration loci of rotational centers or focal origins, with no other ablation, terminated AF in 3.9±3.8 minutes of total ablation to sinus rhythm (n = 13, Fig. 3 and 4), or organized atrial flutter (n = 3). Ablation was typically applied 60–120 minutes after the rotor or focal source was first recorded, further illustrating the temporal stability of human AF sources. Targeted ablation destroyed approximately 2 cm^2^ of tissue to terminate AF, via 5–10 ablation lesions of typical area 0.25 cm^2^, in contrast with conventional ablation for persistent AF that may destroy >12–50 cm^2^ of tissue via 50–200 lesions [34] (each 0.25 cm^2^) or >30% of the area of a 4.6 cm diameter atrium [4] yet terminates persistent AF in fewer than 20% of cases [35]. Specialist centers often aim to achieve AF termination yet, when successful, typically convert AF to atrial tachycardia rather than sinus rhythm [34]. The mechanism by which localized ablation terminates rotors is unclear but likely involves the elimination or alteration of functional or anatomical heterogeneities such as fiber anisotropy [7], [22], [29], fibrosis, scar or other factors [36] central to maintaining AF, leading to its termination. These results demonstrate that targeted ablation of all identified AF sources may terminate fibrillation to normal sinus rhythm in 1–2 orders of magnitude less time and tissue destruction than conventional ablation.

Ablation of rotors or focal beats was successful independent of the type or location of sources. For example, targeted ablation at the rotational center of a left atrial rotor for less than 1 minute converted AF to sinus rhythm (Fig. 3), while targeted ablation of a right atrial rotor terminated AF to sinus rhythm within 5.5 minutes (Fig. 4). Figures 3, 4 and S4 also show clinical electroanatomic shells of the precise ablation lesions applied to terminate AF. Acute termination of AF after localized therapy in only the right atrium is very unusual, and contrasts with conventional ablation which is primarily performed in the left atrium [4]. Our study finds that AF sources often lay in the right atrium (Table S2). The distribution of sources, or the ability of targeted ablation at sources to terminate AF, were unrelated to whether patients were studied for the first time or had previously failed conventional AF ablation.

Ten of the 26 patients had 3 or more organized AF sources, although our protocol permitted ablation at only 2 regions. Localized ablation at 2 sites slowed AF (prolonged cycle length, measured on the coronary sinus channel in routine fashion) by 15±12% in these patients after 6.3±4.3 minutes of targeted ablation. These results show that ablation of all identified localized sources can terminate fibrillation, regardless of the location of the source and whether it is a rotor or a focal beat.

### Targeted ablation results in long-term freedom of AF in patients

After performing targeted ablation, we also performed standard-of-care ablation per our approved protocol (further detailed in Materials and Methods). We then implanted continuous cardiac ECG monitors in 22 of 26 patients to detect recurrent AF, providing more rigorous follow-up than prior studies [4]. Of n = 16 patients with 1–2 localized sources targeted for ablation, 93.8% were continuously free of AF during followup (359±220 days; range: 90 days, blanking, to 861 days) after this single procedure, substantially higher than the single procedure success of conventional ablation [4], [20]. Of n = 10 patients with 3 or more sources, 60% were continuously free of AF during followup (347±272 days; range 90 days, blanking, to 717 days) after this single procedure. Kaplan-Meier curves in figure S5 illustrate these data. These results demonstrate that eliminating all identified rotors or focal impulses sources can improve elimination of AF in the long term.

Our study differs substantially from previous studies using alternative mapping modalities. For example, numerous studies have centered on triggering foci for AF near the pulmonary veins using contact electrodes [37] in populations including those with mitral valve disease [38]. Recent studies have applied the inverse solution to multielectrode arrays within the heart (Ensite 3000, St Jude Medical, Minnesota) [39], [40] or electrodes at the patient's body surface (EcVue™, Cardioinsight, Cleveland, Ohio) [41] to produce maps showing mixed patterns of unstable reentry, focal discharges and less defined waves [31]. Further studies are required using interventions such as ablation to define which of these patterns represent sustaining mechanisms. Contact mapping studies by Allessie and co-workers used high spatial resolution circular electrode plaques (1.8 cm radius) to show transient reentry of meandering wavelets, but covered relatively small atrial regions in patients undergoing surgical valve repair (<15% of the surface area, assuming spherical atria of mean diameter 5.9 cm as reported) [29], [42]. These studies could thus plausibly miss stable sources revealed by our wide field of view mapping, and thus explain why wide-field-of-view mapping revealed localized sources in nearly all patients. Haïssaguerre et al. used careful mapping with a roving catheter to find ‘organized sources’ for AF adjacent to sites of prior unsuccessful ablation [43]. However, our technique revealed sources in all patients (with or without prior ablation), and sources in patients with prior ablation often lay remote from previously ablated left atrial tissue (for instance, in right atrium). Ablation at only rotors and focal sources revealed by our mapping approach (without pulmonary vein isolation) terminated AF predominantly to sinus rhythm in seconds to minutes.

Although electrode separation in our approach is not always equal between splines, this will not alter sequential activation across adjacent electrodes that defines rotational or focal activation. Additional work is required to test whether mitigation of these basket limitations will further improve spatial targeting of rotors or focal sources. Of note, the size of a clinical ablation lesion (5–7 mm) defines the practical resolution required for mapping.

Clinically, while conventional ablation can isolate initiating mechanisms for AF in the pulmonary veins, other initiating mechanisms must remain to explain its suboptimal success [4]. Freedom from AF following a single procedure to directly eliminate AF sustaining sources in our study (81.6%) is substantially higher than after a single procedure to isolate pulmonary veins and other triggers (≈40–50%) in several studies [4], [20]. Moreover, we confirmed success using implanted subcutaneous ECG monitors, that was not used in prior AF ablation trials and is the most rigorous monitoring currently available. Ectopic beats or short-lived AF from initiators may theoretically occur if AF-perpetuating regions alone are destroyed. However, in 2 patients in our study with persistent AF despite prior conventional ablation, targeted ablation at AF sources alone acutely terminated AF and eliminated AF on implanted monitors with no additional ablation. This suggests that the localized sources alone were primary mechanisms sustaining AF in these patients. Further studies are required to identify populations in whom targeted source ablation alone, without conventional ablation, may eliminate AF.

## Conclusions

Our results demonstrate that cardiac fibrillation in human atria, despite its substantial spatiotemporal variability, may be directly caused by very few stable electrical rotors or repetitive focal beats, that are spatially constrained and temporally conserved. Accordingly, limited and rapid ablation was able to terminate fibrillation for long-term elimination. These results were observed in patients with a wide range of AF phenotypes. The demonstration of localized perpetuating sources for human AF using this approach may enable the development of a number of targeted interventions in addition to ablation, including pacing, pharmacologic, gene or regenerative therapies.

## Materials and Methods

### Ethics Statement

The study was approved by the joint Ethics committee of the University of California and Veterans Affairs Medical Centers, San Diego, and written informed consent was obtained from each subject. The authors' responsible joint institutional review board approved the study.

### Patient population

We studied 80 consecutive patients with drug-resistant AF referred for ablation (Table S1), all of whom provided written informed consent for the protocol. The study was approved by our Institutional Review Board (IRB) in two phases (I) n = 54 recruited for procedural collection of data on AF organization; (II) n = 26 in whom intraprocedural mapping enabled targeted ablation at identified AF sources. We enrolled consecutive patients, the only exclusion being an inability or unwillingness to provide informed consent.

### Electrophysiological Data Acquisition

Electrophysiology study was performed after discontinuing anti-arrhythmic medications for >5 half-lives (>60 days after amiodarone). Catheters were advanced to the heart from peripheral veins. After trans-septal puncture, 64-pole basket catheters (Constellation, Boston Scientific, MA) were advanced to the left atrium (all patients) and the right atrium (n = 54 patients) (Fig. 1A–C). Catheters were manipulated carefully to ensure good electrode contact. Electrodes are separated by 4–6 mm along each spline and by 4–10 mm between splines, and thus are able to resolve the ≈40–50 mm minimum reentrant wavelength predicted from minimum human atrial repolarization time (100–110 ms) and slowest dynamic conduction velocity (≈40 cm/s) [30], [44]. Because sources should control activation over a wider area than just the rotor core or focal origin [45], our panoramic approach covers the vast majority of both atria (Fig. 1). This is in contrast to previous contact mapping studies which have a higher spatial resolution but cover far smaller atrial regions [29], [42]. In general, point-by-point (sequential) mapping has focused on electrogram characteristics such as fractionation [46], since spatial maps of AF vary over the timeframe required to complete sequential maps. As discussed above, non-contact mapping techniques have been developed that use mathematical inverse solution approaches to perform global atrial mapping (EcVue™, Cardioinsight, Cleveland, Ohio [41] and Ensite 3000™, St Jude Medical, Minnesota [39], [40]).

Electrode locations were verified within atrial geometry by fluoroscopy and clinical mapping (NavX, St Jude Medical, MN; Fig. 1AB) and enabled creation of spatial maps of normal sinus rhythm (Fig. 1DE) and AF. In phase I, a deflectable 7F monophasic action potential (MAP) catheter (EP technologies, Sunnyvale, CA) was advanced to record MAPs from the right atrium and, via a second trans-septal puncture (performed for clinical ablation), from multiple sites in the left atrium. MAP recordings in AF were used to determine MAP restitution (rate-response) [47] and validate unipolar recordings: activation time intervals that were smaller than the minimum MAP-derived recovery time were discarded.

### Signal Processing

Intracardiac signals were filtered at 0.05–500 Hz, and the ECG at 0.05–100 Hz. Signals were digitized at 1 kHz to 16-bit resolution (Bard Pro, Billerica, MA) for analysis.

We computed spatial activity in AF using 2 approaches. First, we determined AF activation from potentials (electrograms) at each electrode to construct isochronal maps. Second, we performed phase analysis to fibrillation, as first described by Gray et al. [23], by applying the Hilbert transform directly to human unipolar electrograms (Figure S1). Phase analysis can be used to determine spiral wave dynamics in complex computational models [48] and has also been applied to human ventricles [49]. Both approaches led to similar qualitative results. Tip trajectories for each localized source were computed directly from isochronal maps by manual processing, or from phase maps [23]. For the latter, the phase singularity, defined as the point around which the integral of the gradient of the phase did not equate to zero, was assigned periodically throughout multiple cycles. Isolated extreme outlying points representing interpolation errors were excluded. The spatial constraint of rotors (Fig. 2–4) was maintained when data were plotted in raw form or interpolated by third-order Bézier curves, as shown, computed such that first and second derivatives were continuous at the location of the migration locus. In patients undergoing targeted ablation at sources in phase II, the areas of source migration were calculated.

### Ablation to Demonstrate the Mechanistic Role of AF Rotors and Focal Beats

In phase II, targeted ablation was performed directly at AF rotor and focal beat locations to demonstrate their mechanistic role in perpetuating AF in each patient. This is quite distinct to prior mapping studies of AF that generally did not use such interventions to establish whether mapped features were causal or simply associative. Targeted ablation at rotors and focal beats was performed prior to any other intervention. Ablation was performed using an irrigated catheter (Biosense-Webster, Diamond-Bar, CA) at 25–35 W or, in patients with heart failure, a non-irrigated catheter (Boston Scientific, Natick, MA) at 40–50 W, target 52°C. In n = 2 patients with prior conventional ablation in whom targeted ablation terminated AF to sinus rhythm, the pulmonary veins were still isolated and no other ablation was performed. In all other patients, targeted ablation was *followed* by current standard-of-care ablation [4] utilizing wide area circumferential ablation to isolate left and right pulmonary vein pairs with electrical verification of pulmonary vein isolation. Patients with persistent AF also received a left atrial roof line and those with typical atrial flutter received a cavotricuspid isthmus ablation. No other ablation was performed.

To establish that identified AF sources caused the clinical AF phenotype, and not just AF seen acutely in the laboratory, we rigorously followed patients for recurrent AF after targeted elimination of sources. Anti-arrhythmic medications were discontinued after a 3 month blanking period [4], then we used continuous subcutaneous ECG monitoring (84.6% of patients) or external monitors in the remaining 15.4%. No additional ablation was permitted in the blanking period. Freedom from AF was assessed quarterly in clinic for up to 2 years after ablation, and defined as <1% total AF burden. The use of implanted monitors is considerably more rigorous than patient-activated event monitors, ECGs or symptoms alone [4], [50], [51] used in almost all prior AF studies [4].

### Statistical Analysis

Continuous data are represented as mean ± standard deviation (SD). The t-test was used to compare variables between 2 groups. Paired continuous variables were compared using linear regression and the paired *t*-test. Contingency tables were analyzed using the Fisher exact or the Chi-tests when appropriate. A p-value of <0.05 was considered statistically significant. Statistics were calculated using SPSS 19 (IBM, Somers, NY, USA).

## Supporting Information

Table S1 **Patient Characteristics.**
(DOCX)

Table S2 **Characteristics of Human AF Sources.**
(DOCX)

Figure S1 **Phase map of Human Left Atrial Activation During AF.** The phase map was computed using the Hilbert Transform [52] and shows a phase singularity (indicated by the white dot), corresponding to the location of a rotor (same patient as figure 3 of the main manuscript).
(TIF)

Figure S2 **Directionality Analysis of a Rotor During Human Atrial Fibrillation.** **A. shows propagation emanating from the left atrial rotor to the remaining atrium**, in the same patient shown in figure 3 of the main manuscript. The arrows indicate activation direction [53] between isochrones (color bar). **B. Recurrence of predominant direction**, shown as the correlation at each site of the direction over consecutive cycles, showing high recurrence (repeatability) in the annulus adjacent to the rotational center (warm colors) with markedly reduced correlation in a surrounding annulus of tissue (cool colors) with some recovery of repeatability at distant sites.
(TIF)

Figure S3 **Temporal Conservation of a Left atrial rotor in human AF for 237 days.** **A**. Isochronal map of a left atrial rotor obtained prior to conventional ablation that passed outside this source, and did not target it. Atrial fibrillation failed to terminate during ablation, and recurred after the procedure. **B**. Isochronal map of a left atrial rotor at the same location obtained at repeat electrophysiology study 237 days later. Targeted ablation at this source eliminated AF.
(TIF)

Figure S4 **Additional Examples of Brief Targeted Ablation at Stable Sources for Human Atrial Fibrillation.** **A**. Isochronal map of a left atrial focal beat source that lay outside traditional ablation lesion locations. Localized ablation at this site (red dot, and contiguous white dots) terminated AF directly to sinus rhythm in <5 minutes. **B**. Isochronal map of a counterclockwise left atrial rotor on the posterior left atrium near (but not within) the left pulmonary vein antra, where localized ablation (red dots in right panel) terminated AF to sinus rhythm within 3 minutes. Both patients are free of AF on implanted monitors.
(TIF)

Figure S5 **Kaplan-Meier curves for freedom from AF,** detected using rigorous monitoring including implanted continuous ECG recordings.
(TIF)
