# Supplementary material for: Computational Mapping Identifies Localized Mechanisms for Ablation of Atrial Fibrillation
Source: PLoS One. 2012 Sep 26;7(9):e46034. doi: 10.1371/journal.pone.0046034 (PMC3458823; doi:10.1371/journal.pone.0046034)
Supplement: Table S1 — Patient Characteristics. (DOCX) [file pone.0046034.s001.docx]

**Supporting Table 1. Patient Characteristics**

| **Characteristic (n=80)** | **Paroxysmal AF** | **Persistent AF** | **P** |
| --- | --- | --- | --- |
| Age, years | 61±10 | 63±9 | 0.55 |
| Numbers | 26 | 54 (including 6 longstanding) |  |
| Duration of AF, months | 54±74 | 71±65 | 0.64 |
| Left atrial diameter, mm | 42±5 | 48±7 | 0.04 |
| LV ejection fraction, % | 61±8 | 53±13 | 0.30 |
| Coronary Disease, n (%) | 7 (27) | 20 (37) | 0.45 |
| Diabetes Mellitus, n (%) | 8 (31) | 13 (24) | 0.59 |
| Hypertension, n (%) | 19 (73) | 44 (81) | 0.40 |
| Prior Conventional AF ablation, n (%) | 5 (19) | 20 (37) | 0.13 |
| Failed Antiarrhythmic Medications, n (%) |  |  |  |
| Class I | 5 (19) | 12 (22) | 1.0 |
| Sotalol/Dofetilide | 15 (58) | 32 (59) | 1.0 |
| Amiodarone (discontinued > 60 days) | 7 (27) | 30 (56) | 0.02 |
| Other Medications, n (%) |  |  |  |
| ACEI/ARB | 12 (46) | 36 (67) | 0.09 |
| Statins | 14 (53) | 34 (63) | 0.47 |
| Beta-blockers | 16 (62) | 38 (70) | 0.45 |
