# Supplementary material for: Computational Mapping Identifies Localized Mechanisms for Ablation of Atrial Fibrillation
Source: PLoS One. 2012 Sep 26;7(9):e46034. doi: 10.1371/journal.pone.0046034 (PMC3458823; doi:10.1371/journal.pone.0046034)
Supplement: Table S2 — Characteristics of Human AF Sources. (DOCX) [file pone.0046034.s002.docx]

**Supporting Table 2. Characteristics of Human AF Sources**

| **Characteristic** | **(Longstanding) Persistent AF (n=54)** | **Paroxysmal AF (n=26)** | **P** |
| --- | --- | --- | --- |
| AF Cycle Length, (average, SD; ms)  Left Atrial Appendage  Left Atrial Mean | 169±22  179±26 | 192±24  207±37 | <0.001  <0.001 |
| Patients with sources (%) | 51 (94) | 26 (100) | NS |
| No. Chambers mapped, Left/Right atria | 54/38 | 26/16 |  |
| No. Rotors, Left/Right Atrium | 46/34 | 17/6 |  |
| No. Focal Beats in Left/Right Atrium | 17/1 | 12/3 |  |
| Source Cycle Length, (mean±SD; ms) |  |  | 0.01 |
| Rotors, Left/Right Atrium | 162±18/173±26 | 182±24/179±17 |  |
| Focal Beats, Left/Right Atrium | 195±34/183 | 207±21/217±38 |  |
| No. Co-existing Sources (rotors/focal beats) (in patients with bi-atrial recordings) | 2.0±0.8 | 1.5±0.8 | <0.01 |
| Source Migration Area of Migration (cm^2^) (measured in phase II of study) | 2.5±1.4 | 2.2±1.0 | NS |
