# Supplementary figures and images for: Computational Mapping Identifies Localized Mechanisms for Ablation of Atrial Fibrillation
Source: PLoS One. 2012 Sep 26;7(9):e46034. doi: 10.1371/journal.pone.0046034 (PMC3458823; doi:10.1371/journal.pone.0046034)

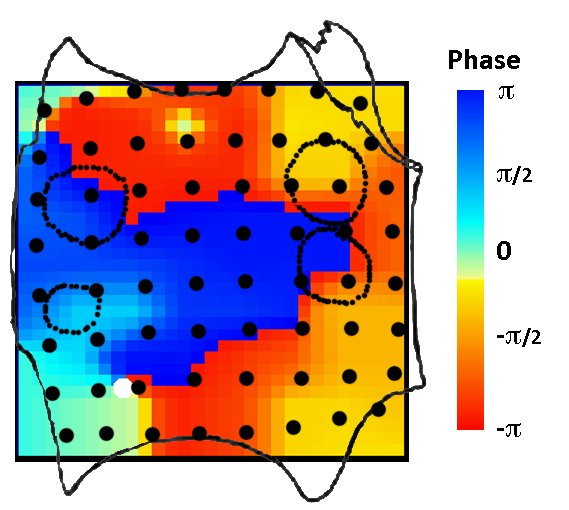

Supplement: Figure S1 — Phase map of Human Left Atrial Activation During AF. The phase map was computed using the Hilbert Transform [52] and shows a phase singularity (indicated by the white dot), corresponding to the location of a rotor (same patient as figure 3 of the main manuscript). (TIF) [file pone.0046034.s003.tif]

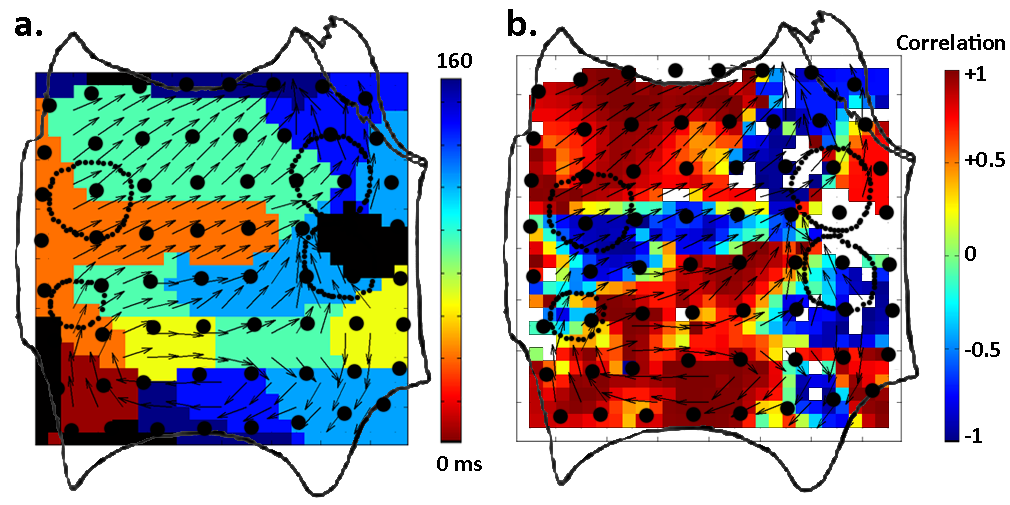

Supplement: Figure S2 — Directionality Analysis of a Rotor During Human Atrial Fibrillation. A. shows propagation emanating from the left atrial rotor to the remaining atrium, in the same patient shown in figure 3 of the main manuscript. The arrows indicate activation direction [53] between isochrones (color bar). B. Recurrence of predominant direction, shown as the correlation at each site of the direction over consecutive cycles, showing high recurrence (repeatability) in the annulus adjacent to the rotational center (warm colors) with markedly reduced correlation in a surrounding annulus of tissue (cool colors) with some recovery of repeatability at distant sites. (TIF) [file pone.0046034.s004.tif]

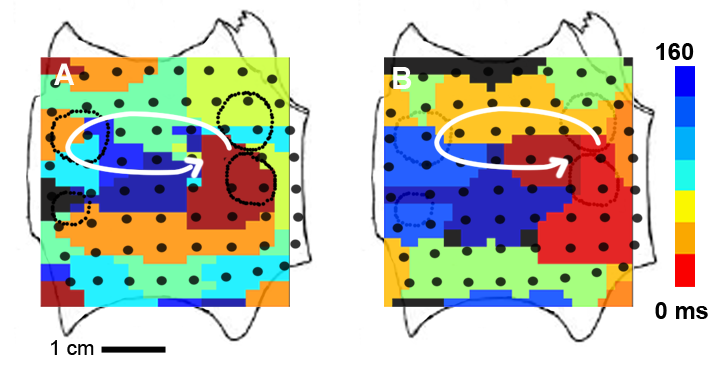

Supplement: Figure S3 — Temporal Conservation of a Left atrial rotor in human AF for 237 days. A. Isochronal map of a left atrial rotor obtained prior to conventional ablation that passed outside this source, and did not target it. Atrial fibrillation failed to terminate during ablation, and recurred after the procedure. B. Isochronal map of a left atrial rotor at the same location obtained at repeat electrophysiology study 237 days later. Targeted ablation at this source eliminated AF. (TIF) [file pone.0046034.s005.tif]

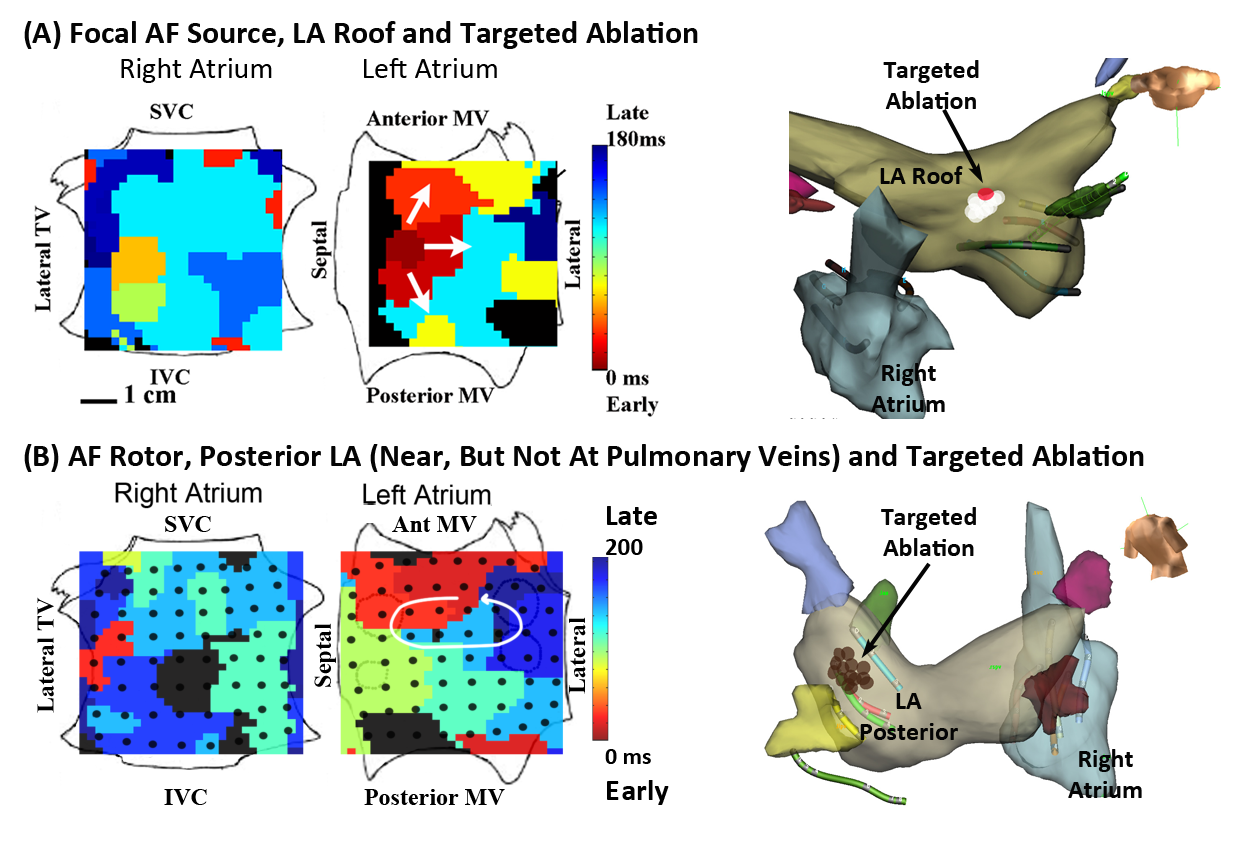

Supplement: Figure S4 — Additional Examples of Brief Targeted Ablation at Stable Sources for Human Atrial Fibrillation. A. Isochronal map of a left atrial focal beat source that lay outside traditional ablation lesion locations. Localized ablation at this site (red dot, and contiguous white dots) terminated AF directly to sinus rhythm in <5 minutes. B. Isochronal map of a counterclockwise left atrial rotor on the posterior left atrium near (but not within) the left pulmonary vein antra, where localized ablation (red dots in right panel) terminated AF to sinus rhythm within 3 minutes. Both patients are free of AF on implanted monitors. (TIF) [file pone.0046034.s006.tif]

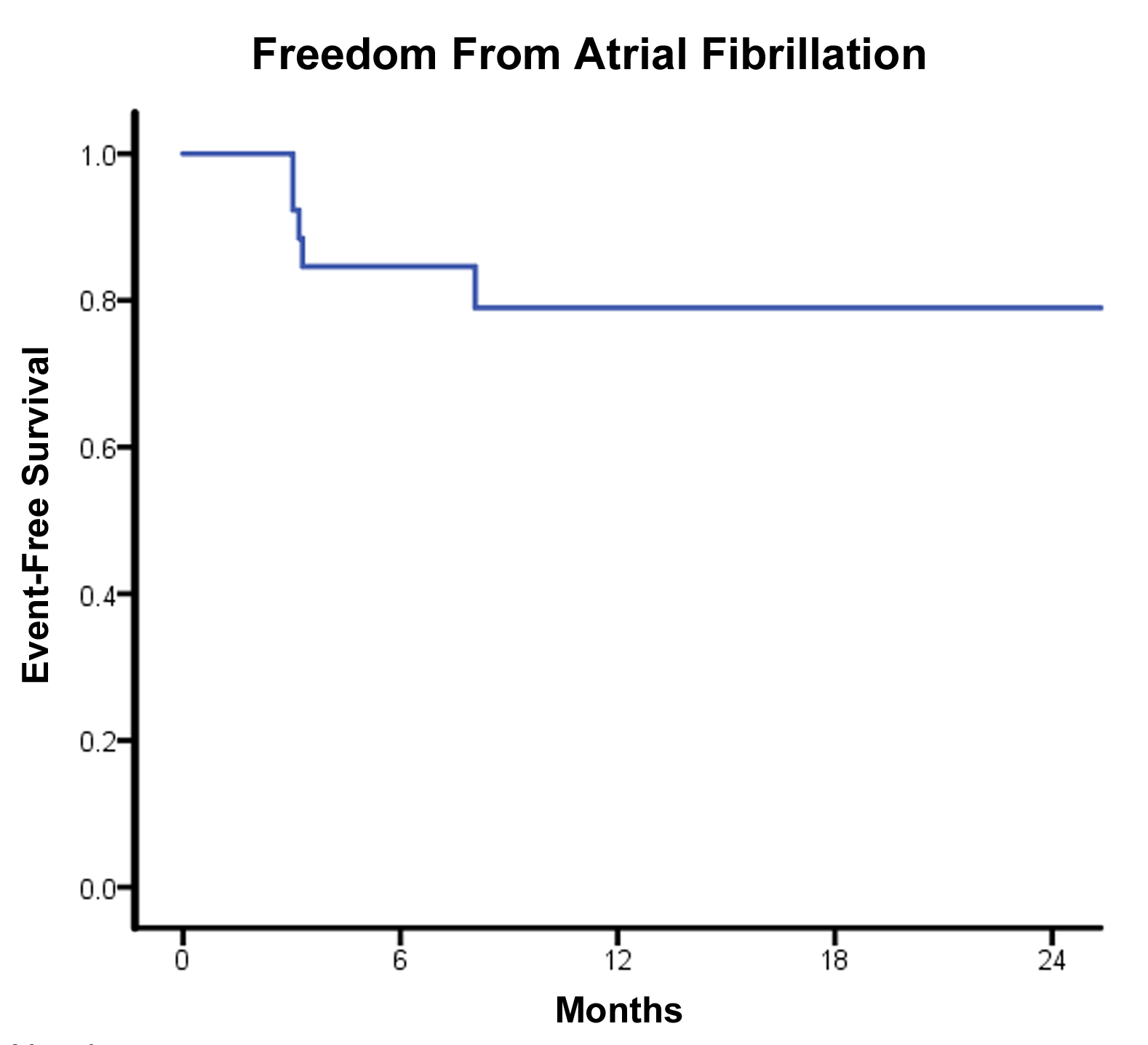

Supplement: Figure S5 — Kaplan-Meier curves for freedom from AF, detected using rigorous monitoring including implanted continuous ECG recordings. (TIF) [file pone.0046034.s007.tif]
